# An investigation of cognitive test performance across conditions of silence, background noise and music as a function of neuroticism

**DOI:** 10.1080/10615806.2013.864388

**Published:** 2013-12-10

**Authors:** James Reynolds, Alastair McClelland, Adrian Furnham

**Affiliations:** Research Department of Clinical, Educational and Health Psychology, University College London, London, UK

**Keywords:** personality, neuroticism, distraction, test performance

## Abstract

The present study investigates the role of trait neuroticism on cognitive performance under distraction. Seventy participants were given a personality test and then undertook a number of different cognitive tasks in silence, in the presence of popular music and in background noise. It was predicted that performance on a general intelligence test, a test of abstract reasoning, and a mental arithmetic task would be adversely affected by background sounds. It was predicted that neuroticism would be negatively correlated with performance on the mental arithmetic task but only when the individuals were working in the presence of background sound. Stable vs. unstable participant's performance on a mental arithmetic task during noise was significantly higher as predicted. The results provided partial support for the hypotheses and are discussed with respect to previous findings in the literature on personality (particularly introversion-extraversion) and distraction on cognitive task performance. Limitations are noted.

## Introduction

In contemporary society, listening to music while conducting daily activities is commonplace. Indeed, Ransdell and Gilroy (2001) reported that the majority of college students listen to music while working on a computer. Moreover, background music (typically known as “piped-in” music) is popular within numerous organizations, including restaurants, offices, and hospitals (Bitner, 1992) with the majority of people employed in such organizational settings also having access to music through devices such as MP3 players and the Internet (Bull, 2005). Due to the ubiquity of background sound (both music and noise) in applied settings, the effects of both types of sound on cognitive test performance have been investigated. This is a highly active research area (Gordon-Hickey & Lemley, 2012; Patston & Tippett, 2011), but fewer studies have looked at individual differences in this process.

The research on when and why background sound affects productivity has tended to focus on three elements (Hoch, Poulin-Charronnat, & Tillmann, 2011; Huang & Shih, 2011; Jancke & Sandmann, 2010; Shih, Huang, & Chiang, 2009; Smith, Baranski, Thompson, & Abel, 2003). These are the *nature of the distraction*, for instance, noise vs. music, *task demands* (simple vs. complex), and *personality* differences – in particular high vs. low extraversion (Banbury & Berry, 1998). To some extent these differences are not that clearly defined: one person's music is another's noise; a simple task for one person is complex for another. This study looks at all these factors focusing on trait neuroticism, which has been neglected in comparison with other traits, particularly extraversion.

Early studies have illustrated that music is more likely to have an effect on performance when individuals perform complex performance tasks (Kirkpatrick, 1943; McGhee & Gardner, 1949). Williams (1961) reported that classical music had no effect on reading comprehension while Mammarella, Fairfield, and Cornoldi (2007) demonstrated that digit span and phonemic fluency tasks were better performed in the presence of Vivaldi than in silence.

Lesiuk (2005) found that that state positive affect and work quality were higher in the presence of music, while time-on-task was shorter when music was present indicating that the participants spent more time on a task than intended when music was absent. It should be noted that in this quasi-experimental study, the participants could choose which type of music they wanted to listen to, and were also in control of the length of time they listened. Perham and Sykora (2012) found that performance on a serial recall task was more adversely affected by music the participants liked that by music they disliked. Performance in silence was superior to both music conditions. However, there was less acoustical variation in the disliked than the liked music – and variation is known to have a disruptive effect on serial recall but not on tasks that do not require the process of seriation (Beaman & Jones, 1997).

Beaman (2005, p. 1059) reviewed the literature concerning the distracting effect of noise on task performance at work, and noted that noise which is characterized by “abrupt changes in frequency or pitch is particularly disruptive” and much more disruptive than changes in intensity. The effects of noise can also be task specific; for example, Banbury and Berry (1998, Expt. 1) found that background noise impaired performance on a mental arithmetic task, but not a memory for prose task, whereas background noise plus speech had a detrimental effect on performance in both tasks. The distraction/task effect is usually explained in terms of task impairment being caused by a conflict in the processing of the task (Marsh, Hughes, & Jones, 2008).

The effects of differences in personality on cognitive test performance have also been investigated. This is the main focus of this paper. There are many important papers showing how personality traits relate to information processing (Bates & Rock, 2004; Revelle, Amaral, & Turriff, 1976). This literature has recently been reviewed by von Stumm, Chamorro-Premuzic, & Ackerman (2011). Results have shown that test performance is negatively correlated with neuroticism (Zeidner & Matthews, 2000), positively correlated with conscientiousness (Moutafi, Furnham, & Crump, 2003), and openness to experience (Chamorro-Premuzic, Moutafi, & Furnham, 2005) and both positively and negatively correlated with extraversion (Ackerman & Heggestad, 1997).

Individuals who score high on neuroticism are characterized as being emotional, moody, sensitive, frequently depressed, and suffering from numerous psychosomatic disorders (Eysenck & Eysenck, 1975). In contrast, individuals who score low on neuroticism are characterized as having a secure and hardy temperament, and being generally relaxed even under adverse conditions. Eysenck (1967) proposed that neuroticism is mediated by the levels of arousal within the limbic system, and suggested that individual differences arise because of differences in activation level between individuals. He also suggested that highly neurotic people who are presented with minor stressors will exceed optimal levels of arousal in comparison to those individuals low in neuroticism.

There is now an extensive literature on the effects of background noise and music on the cognitive test performance of introverts and extraverts (Furnham & Strabc, 2002; Seo, Hahner, Gudziol, Scheibe, & Hummel, 2012). For instance, Dobbs, Furnham, and McClelland (2011) illustrated how introverts performed less well on a number of tasks than extraverts in the presence of background noise and music but in silence performance was the same.

The trait of neuroticism under such conditions has been less extensively investigated. Indeed, it seems only Dobson (2000) looked at the effect of neuroticism (and extraversion) on (cognitive) selection test performance. He showed that increased neuroticism is associated with poorer test performance on a numerical reasoning task (but not verbal reasoning) under conditions of stress – and this relationship is not evident under more relaxed conditions. This finding suggests that the performance of individuals high in neuroticism will be underestimated if the testing conditions lead to test anxiety and the task places a heavy demand on information processing capacity. In the present study, we investigate both the effect of neuroticism and background sound (silence, music, and noise) on performance across four cognitive tests; the Wonderlic Personnel Test (WPT) (general cognitive ability), Baddeley's sentence-checking task (verbal fluency), a mental arithmetic test, and Raven's advanced progressive matrices (abstract reasoning).

We predicted that performance on the WPT and Raven's progressive matrices would be better under conditions of silence than in the presence of music or noise (Dobbs et al. 2011) (H1). We also predicted that performance on the mental arithmetic task would be adversely affected by noise (Banbury & Berry, 1998, Expt. 1) (H2a) but that the sentence-checking task would be unaffected (Baddeley, 1968) (H2b). Finally, we anticipated that degree of neuroticism would be negatively related to task performance on the mental arithmetic task – but only under conditions in which participants were stressed (Dobson, 2000) (H3). Thus, in the context of the current study, we predicted a negative relationship between neuroticism and task performance in the presence of a stressor (distracting sound) but no relationship between neuroticism and performance in the silent condition.

## Methods

### Participants

A convenience sample of 70 students (26 males and 44 females) aged 16–18 (*M* = 16.71 years, SD = 0.68 years) were recruited from a London (UK) college.

### Materials

#### Tests administered

(1) The WPT is 12 minutes long in duration. It consists of 50 items graded in difficulty testing problem solving using a range of algebraic and geometric techniques. It is a test of general cognitive ability (fluid and crystallized intelligence) and has a high correlation with the Wechsler Adult Intelligence Scale (Wonderlic Personnel Test Inc., 1992). Items include number and word comparisons where participants have to write down the correct answer.
(2) Baddeley's (1968) sentence-checking test is a 64 question test administered in 3 minutes measuring verbal fluency. Participants were presented with a series of sentences, each describing presentational order of two letters, A and B. Each sentence was followed by either pair AB or BA, and participants were required to decide whether the sentence correctly described the attached letter pair. The test is extensively used as a very quick measure of fluid intelligence (Furnham & McClelland, 2010).
(3) The mental arithmetic test was adapted from Lock (2008). Participants had to answer as many questions out of 30 as they could within a 15-minute time period. Each item had 10 procedures: 8, × 6, +1, /7, × 5, Double it, +2, /9, Times by itself, −9, =??. There are three versions: beginner, intermediate, and advanced. This group has examples from the intermediate tests.
(4) Raven's advanced progressive matrices is a nonverbal reasoning measure. It tests abstract reasoning and consists of 36 items. Within each item contains a figure which is missing a piece, and below are eight options where one option must be selected to complete the figure. Participants completed as many as possible in 15 minutes.
(5) The NEO-Five Factor Inventory (NEO-FFI) (McCrae & Costa, 2004) was used to assess neuroticism. It consists of 60 items (12 per scale) designed to take approximately 10–15 minutes to administer. An example of neuroticism: “I rarely feel fearful or anxious.” Participants responded on a five-point scale from “strongly disagree” to “strongly agree,” and each individual's score for each trait was found by summing scores for the 12 items. It is considered a highly valid measure of all big five traits.

#### Sounds

Two digital voice recorders were used. One recorded background noise stimuli and the other music stimuli. Both were played at 70 db. Background sound consisted of samples including police cars, construction work, and air raid sirens as well as sounds found in everyday working environments. The finished length of the combined noises was 15 minutes and 14 seconds.

The music compositions selected included contemporary dance remixes to reflect interests of the target demographic tested; they are likely to have recognized the chosen tracks as they are frequently heard in the charts and on different radio stations. The three compositions selected all had a fast tempo and vocals throughout. Vocals are usually thought of as more distracting than music without words. The songs chosen were Changed The Way You Kissed Me by Example (Mensah Remix), What A Feeling by Alex Gaudino featuring Kelly Rowland (Nicky Romero Remix), and Louder by DJ Fresh featuring Sian Evans (Drumsound & Bassline Smith Remix). The finished length of combining all three tracks was 15 minutes and 1 second.

### Design

A Latin Square design was used, and each of the tests was completed under conditions of silence, noise, and music. This was achieved by splitting participants into three approximately equal groups (a 24/23/23 split) with each group performing the same tests but with a different mapping of sound conditions to tests. The independent variables were sound condition (silence, noise, or music) and degree of neuroticism, and the dependent variables were measures of performance on the four cognitive tests.

### Procedure

Permission from the college was obtained, with participants randomly allocated to one of the three groups. Each participant was seated, so other participant's answers could not be seen and each was encouraged to keep their eyes on their own work. Each participant was given the NEO-FFI at the start of the investigation. Every participant then completed each task; either under conditions of silence, noise, and music. Upon completion of each task, each of the three groups switched to a different sound condition (see the Latin Square design). The background noise and music were played from the two digital voice recorders which were placed at the front of the rooms and maintained at a constant volume level during the study. Participants were given 15 minutes to complete each task – with the exception of the WPT and Baddeley's sentence-checking test. As these tasks could be completed more quickly, they were combined into one test session – which lasted 15 minutes in total. Within the session, the tasks were counterbalanced to control for possible order effects. The mental arithmetic test and Raven's advanced progressive matrices made up the remaining two conditions. Participants completed as many of the items as they could for each test within the time allowed. In all testing took around one hour.

## Results

The correlations between neuroticism and performance on the tests are presented in Table 1.

**Table 1.** Table of correlations between neuroticism and tests of cognitive ability.

	Neuroticism	WPT	Sentence-checking	Mental arithmetic
WPT	0.003			
Sentence-checking	−0.151	0.259*		
Mental arithmetic	−0.283*	0.314**	0.142	
Raven's	−0.145	0.165	0.028	0.155

Note: *p < .05, **p < .01.

Neuroticism was significantly negatively correlated with performance on the mental arithmetic test, but did not correlate with any other test. Usually correlations are around *r* = .10 (Poropat, 2009). WPT performance was significantly positively correlated with performance on both the sentence-checking task and the mental arithmetic task – although the correlations are modest. Performance on Raven's did not correlate with any of the other measures. To avoid the loss of statistical power associated with dichotomizing quantitative variables (MacCallum, Zhang, Preacher, & Rucker, 2002) rather than using a median-split to create groups of participants either high or low on neuroticism, a series of multiple regressions were conducted. For each cognitive test, a model was constructed with background sound (dummy coded) as a categorical variable, and neuroticism and a continuous variable. An interaction term between background sound and neuroticism was also included. Prior to each analysis, the neuroticism variable was centered, so that effect of background sound could be evaluated at the mean level of neuroticism (i.e., a comparison of means adjusted for neuroticism). These means are presented in Table 2.

**Table 2.** Table of adjusted means and standard errors for the Wonderlic Personnel Test, sentence-checking, mental arithmetic, and Raven's under the conditions of silence, music, and noise.

	Condition		
Test	Silence	Music	Noise
*Wonderlic Personnel Test*			
*M*	21.46	13.97	7.61
SE	0.91	0.92	0.93
*Sentence-checking*			
*M*	22.47	16.42	17.91
SE	2.20	2.22	2.24
*Mental arithmetic*			
*M*	8.34	8.70	6.89
SE	0.97	0.95	0.96
*Raven's*			
*M*	11.37	8.75	9.37
SE	1.01	1.02	1.00

### Wonderlic Personnel Test

**Figure 1.**
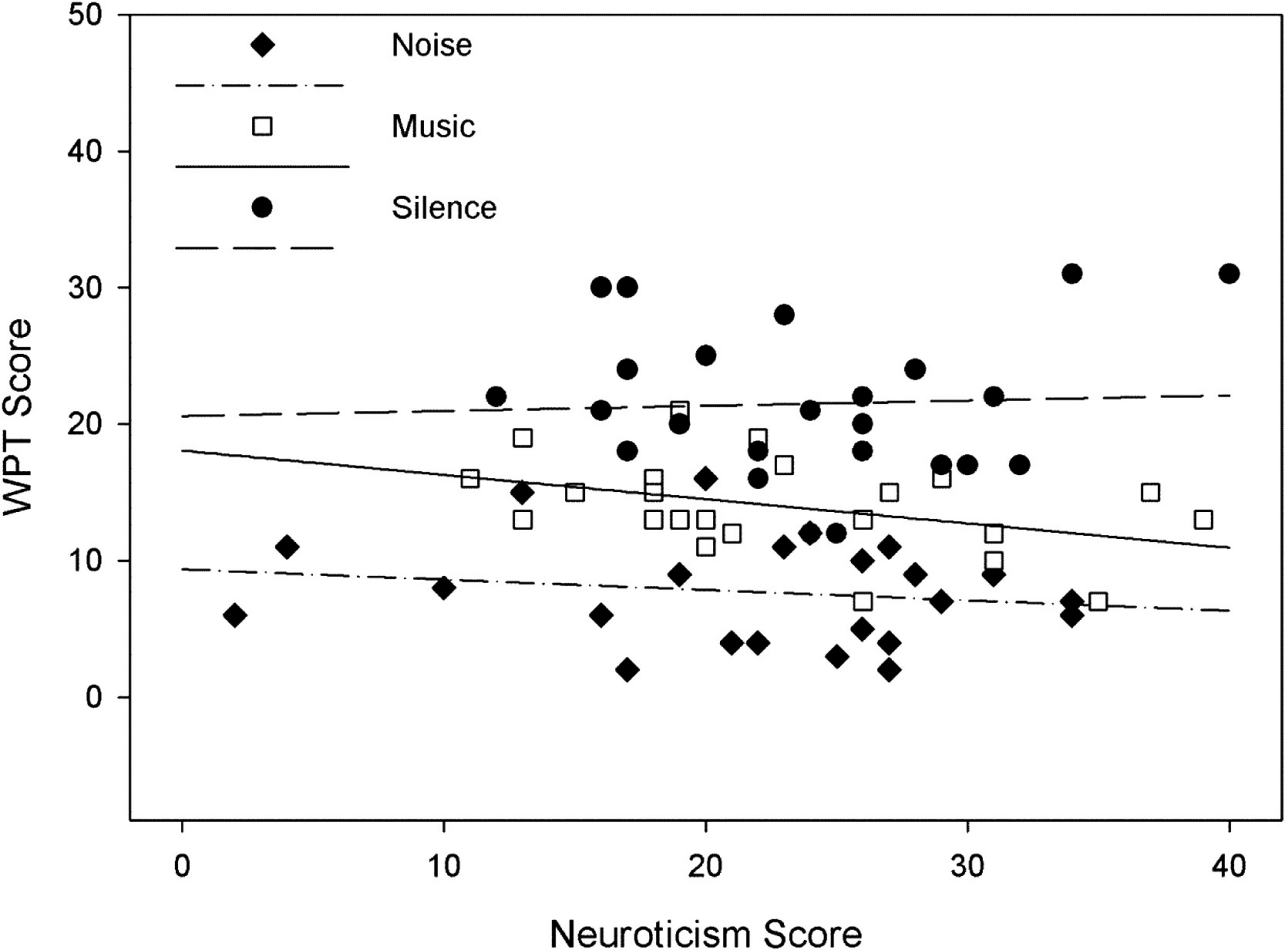
Performance on the WPT as a function of background sound condition and neuroticism.

The relationship between performance on the WPT, background sound condition, and neuroticism is shown in Figure 1.

With respect to background noise, planned orthogonal contrasts revealed that performance in silence (*M* = 21.46) was significantly better than in the presence of sound (*M* = 10.79), *F*(1, 64) = 91.26, *p* < .001, η^2^_*p*_ = 58.8%, and performance in the presence of noise was significantly worse than in the presence of music, *F*(1, 64) = 34.65, *p* < .001, η^2^_*p*_ = 27.4%. Furthermore, non-orthogonal pairwise contrasts showed that performance in silence was better than with music and with noise (*p* < .001 in both cases). There was no significant effect of neuroticism on WPT performance, *F*(1, 64) = 1.02, *p* = .316, η^2^_*p*_ = 1.6% and no significant interaction between background sound and neuroticism, *F* < 1.

### Baddeley's sentence-checking test

**Figure 2.**
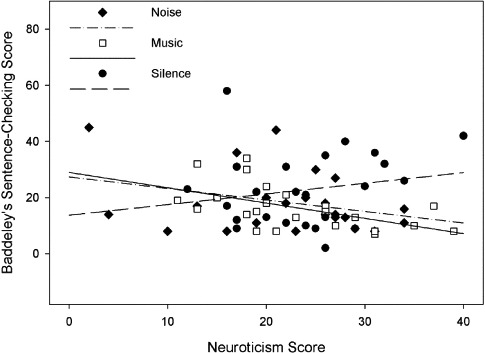
Performance on Baddeley's sentence-checking test as a function of background sound condition and neuroticism.

The relationship between performance on the Baddeley's sentence-checking task, background sound condition, and neuroticism is shown in Figure 2.

Planned comparisons revealed no significant effect of background noise on test performance. The difference between performance in silence (*M* = 22.47) and in the presence of sound (*M* = 17.13) just failed to reach significance, *F*(1, 64) = 3.90, *p* = .053, η^2^_*p*_ = 5.7%, but there was no significant difference in performance between the music and noise conditions, *F* < 1. There was no main effect of neuroticism, *F*(1, 64) = 1.24, *p* = .270, η^2^_*p*_ = 1.9%, and the interaction between background sound and neuroticism also failed to reach significance, *F*(2, 64) = 2.48, *p* = .092, η^2^_*p*_ = 7.2%.

### Mental arithmetic test

**Figure 3.**
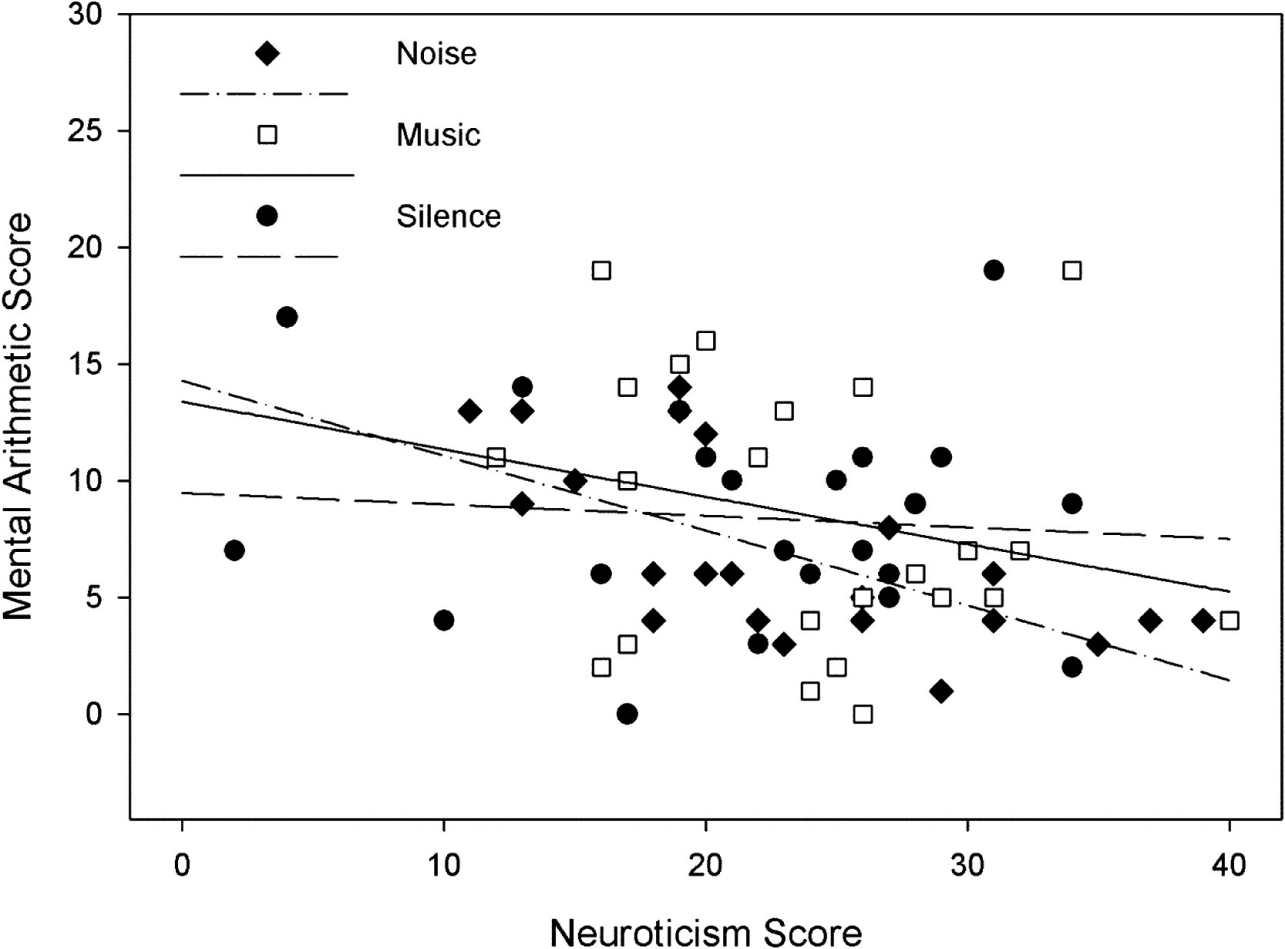
Performance on the mental arithmetic test as a function of background noise condition and neuroticism.

The relationship between performance on the mental arithmetic test, background sound condition, and neuroticism is shown in Figure 3.

No significant effect of background sound was evident for this task, as neither of the orthogonal planned contrasts were significant; silence vs. sound, *F* < 1, and music vs. noise, *F*(1, 64) = 1.93, *p* = .19, η^2^_*p*_ = 2.9%. However, there was a significant effect of neuroticism on test performance, *F*(1, 64) = 6.57, *p* = .013, η^2^_*p*_ = 9.3%. Although the interaction between background sound and neuroticism was not significant, *F*(2, 64) = 1.27, *p* = .287, η^2^_*p*_ = 3.8%, simple effects analysis revealed that although degree of neuroticism was not significantly related to performance under conditions of silence, *F* < 1, or music, *F*(1, 22) = 1.32, *p* = .263, η^2^_*p*_ = 5.7%, it was strongly related to performance under noise, *F*(1, 21) = 15.12, *p* < .001, η^2^_*p*_ = 41.9%. Correlational analysis revealed that there was no correlation between neuroticism and performance in silence (*r* = −.09), a weak negative correlation in the music condition (*r* = −.24) and a strong negative relationship found between these measures in the noise condition (*r* = −.65 – as shown in Figure 3).

### Raven's advanced progressive matrices

**Figure 4.**
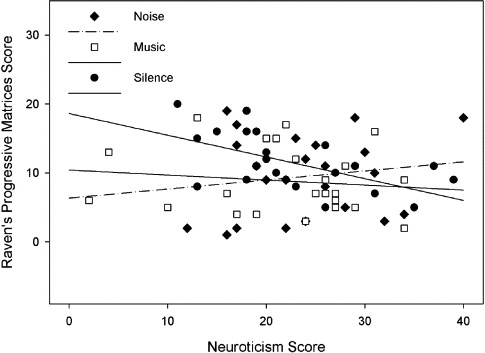
Performance on Raven's progressive matrices as a function of background sound condition and neuroticism.

The relationship between performance on Raven's progressive matrices, background sound condition, and neuroticism is shown in Figure 4.

There was no significant difference between performance in silence and with sound, *F*(1, 64) = 3.17, *p* = .08, η^2^_*p*_ = 4.7%, nor between music and noise (*F* < 1). Degree of neuroticism also had no effect on test performance, *F*(1, 64) = 1.19, *p* = .279, η^2^_*p*_ = 1.8%, and there was no significant interaction between the variables, *F*(2, 64) = 2.51, *p* = .089, η^2^_*p*_ = 7.3%.

## Discussion

The aim of the current investigation was to further extend the research on the effects of background sound and personality on cognitive test performance. The results were mixed for some tasks previous findings were replicated and extended or at least partially replicated; for other findings this was not the case.

Performance on the Wonderlic test was adversely affected by music, and even more so by noise, thus replicating the findings from Dobbs et al. (2011). However, these authors also found a clear effect of background sound on performance for Raven's matrices – but in the present study, there was no indication that performance was adversely affected by noise, and rather weak evidence that it was affected by music. Baddeley (1968) reported that performance on his sentence-checking task was unaffected by loud white noise, and we also found no effect of noise on performance in the current study. However, there was weak evidence to suggest that performance may have been adversely affected by music – a stressor not investigated by Baddeley. The prediction that performance on the mental arithmetic task would be worse in noise than in silence was not supported, but we did find that degree of neuroticism was strongly and inversely related to performance on this task when the participants were subjected to noise, but not – as we predicted – in the presence of music. There was no relationship between test performance and neuroticism in conditions of silence.

The negative correlation between neuroticism and mental arithmetic can in the main be attributed to the strong relationship found between these measures in the noise condition. However, there was a weak correlation for the music condition and although the relationship was not significant, the results suggest that with greater statistical power a relationship between distraction by music and neuroticism might have been evident – but weaker than that between noise and neuroticism. These findings replicate and extend those of Dobson (2000) who found a negative relationship between performance on a numerical reasoning test and neuroticism – but only when the participants were taking a selection test for a place on an MBA program. Dobson concluded that neuroticism undermines cognitive performance under stressful test conditions, and thus scores obtained under such circumstances are likely to underestimate the true abilities of the individuals concerned. The present study indicates that simply placing neurotic individuals in noisy conditions can have the same adverse effect on some task performances.

Dobson (2000) found no relationship between degree of neuroticism and performance on a verbal reasoning task. He suggested that the verbal reasoning task was less demanding in terms of information processing capacity this made it less susceptible to interference, and speculated that “This may be because verbal reasoning processes are more practised and therefore require less processing capacity” (p. 108). In the present study, there was no evidence of a relationship between neuroticism and performance on the Baddeley (1968) sentence-checking task – or indeed any of the other cognitive tests investigated. Thus, the negative relationship between test performance and degree of neuroticism appears to be restricted to arithmetical/numerical reasoning tasks.

These results can also be interpreted in terms of the study of Moutafi, Furnham, and Tsaousis (2006) who induced state test anxiety in students when completing an intelligence test. They found that neuroticism was related to intelligence test scores but only for the high-anxiety group. That is, whereas neuroticism was not correlated with performance once state anxiety was partialled out. In that study anxiety was induced by instructions while in this study they were induced by musical distraction.

With respect to the effects of distraction per se, perhaps the most puzzling finding in the present study was the lack of an effect of background sound on Raven's progressive matrices. Dobbs et al. (2011) found a negative effect of both music and noise on Raven's performance, but in the present study, although performance was best in silence, it was only in the music condition that there was weak evidence for an adverse effect of the distractor. Indeed, in three of the four tasks (mental arithmetic being the exception), average performance was highest in the silent condition and the failure to observe the detrimental effects of background sound may, at least in some instances, be attributed to a lack of statistical power. That said, the failure to find a main effect of background sound on the mental arithmetic task replicates the result obtained by Furnham and Strabac (2002).

This test had limitations which concerned all three factors investigated. We measured neuroticism rather than a direct measure of trait anxiety which may have yielded different results. Next, our measure of noise may not have been representative of most everyday background sounds and had more acoustical variation than is common. Third, perhaps most important for this area was the choice of cognitive performance tasks which were not sufficiently differentiated with one involving only words (Baddeley), one numbers (mental arithmetic) and one shapes (Ravens) and one all three (WPT). While the focus was on personality the other two factors (task and sound) warrant clearer identification and choice to see meaningful interactions. Nevertheless, this represents a useful step in an under-researched area in applied ergonomics.
